# Polymer Matrix Nanocomposites Fabricated with Copper Nanoparticles and Photopolymer Resin via Vat Photopolymerization Additive Manufacturing

**DOI:** 10.3390/polym16172434

**Published:** 2024-08-28

**Authors:** Leon D. Gil, Sergio Neves Monteiro, Henry A. Colorado

**Affiliations:** 1 CCComposites Laboratory, University of Antioquia, Calle 67 No. 53–108, Medellín 050010, Colombia; leon.gil@udea.edu.co; 2 Military Institute of Engineering, IME, Praça General Tibúrcio 80, Urca, Rio de Janeiro 22290-270, RJ, Brazil; snevesmonteiro@gmail.com

**Keywords:** nanocomposites, nanoparticles, particle-reinforcement, vat photopolymerization, copper

## Abstract

This investigation explores the fabrication of polymer matrix nanocomposites via additive manufacturing (AM), using a UV photopolymerization resin and copper nanoparticles (Cu-NPs) with vat photopolymerization 3D printing technology. The aim in this study is to investigate the mentioned materials in different formulations in terms of inexpensive processing, the property related variability, and targeting multifunctional applications. After the AM process, samples were post-cured with UV light in order to obtain better mechanical properties. The particles and resin were mixed using an ultrasonicator, and the particle contents used were 0.0, 0.5, and 1.0 wt %. The process used in this investigation was simple and inexpensive, as the technologies used are quite accessible, from the 3D printer to the UV curing device. These formulations were characterized with scanning electron microscopy (SEM) to observe the materials’ microstructure and tensile tests to quantify stress–strain derived properties. Results showed that, besides the simplicity of the process, the mixing was effective, which was observed in the scanning electron microscope. Additionally, the tensile strength was increased with the UV irradiation exposure, while the strain properties did not change significantly.

## 1. Introduction

Additive manufacturing (AM), also known as 3D printing (3DP), is the process of creating parts layer by layer [1]. In this process, a digital design is translated into a framework that an additive manufacturing device uses to then fabricate a real 3D object, either from a liquid, powdered, or solid raw material, unlike conventional subtracting manufacturing, where material is removed from a solid initial piece. The versatility of AM has revolutionized the way objects and parts are created in almost all technological fields, spanning from transportation to biomedical applications [2]. AM has been used for almost all materials and complex shapes, not only showing no limits and versatility to new challenges [3], but also improving sectors as diverse as electronics materials and devices [4]; aerospace components and space exploration [5,6]; construction and building materials [7,8]; pollution reduction [9,10]; education and learning strategies [11]; food [12]; biomedicine [13,14]; and armor and military applications [15,16]. Additive manufacturing is revolutionizing businesses as well as facilitating innovation and startups, as many of the technologies are very inexpensive and available elsewhere. AM can be classified into seven technologies according to the ISO/ASTM 52900 standard [17]: vat polymerization, sheet lamination, powder bed fusion, material jetting, material extrusion, directed energy deposition, and binder jetting. These technologies can not only be used for engineering materials, but also can be used, as mentioned, for others, including food [12] and natural fibers composites [18,19,20].

Among these techniques, vat photopolymerization is a very interesting methodology because it allows the manufacturing of polymer-based materials with a high level of accuracy and good finish, at relatively high speeds and reasonably low cost.

The photopolymerization process is completed over a light-curable, photopolymer resin, which is stored in a vat container and typically exposed to UV light irradiation. The light causes the polymeric resin to cure, through an irreversible polymerization reaction, in which, upon light exposure, the photo-initiators release species that work as catalysts, creating chains from monomers and oligomers [21]. Three common methods of vat photopolymerization are stereolithography (SLA) cured with a laser, digital light processing (DLP) cured with a projector, and continuous digital light processing (CDLP) cured with LEDs and oxygen. All these methods allow final parts to have intricate details at very small scales and excellent surface finishing. Vat photopolymerization has a wide variety of applications in fields such as aerospace, automotive, and medical industries due to its superior mechanical strength and dimensional accuracy [22]. When making composites or nanocomposites with this technology, one of the challenges is to produce a well dispersed distribution of the nano- or micro particles or fibers added. Moreover, the addition of solids can negatively affect other properties such as surface finishing, homogeneity, curing parameters, and porosity, which can be tailored to improve each material’s formulation and application [23]. Recent nanocomposite materials manufactured using vat photopolymerization methods exhibit improved strength, thermal stability, and electrical conductivity, among other properties.

Nanocomposites are materials that involve at least one dimension or component of less than 100 nm [24,25], and are characterized by enhanced mechanical, thermal, electronic, and many other properties when compared to micro and macro materials, which are mainly associated with the almost-zero crystalline defects. Polymer nanocomposites [26] are the most fabricated among the nanocomposites, constituted typically by a polymeric binder (thermoset, thermoplastic, or elastomer) with nanoparticles or nanotubes.

Vat photopolymerization (VPP) methods have been used intensively in recent years to produce polymer nanocomposites [27]. One area of active research is the development of formulations with improved mechanical properties, such as increased strength, toughness, and stiffness, with the incorporation in most cases of ceramic nano-reinforcements [28]. A significant part of the available research shows development in other properties and functional materials. For instance, carbon nanotubes (CNTs) have been shown to decrease and control the photocurable properties of the resin in the VPP process [29], contributing to the process optimization. Other works show that CNTs contribute to reduced anisotropy in conductive polymers made with VPP [30], which is reduced by almost two orders of magnitude, enabling uniform electronics. Graphene is another nano-structural material used in VPP process, with applications in electronics, aerospace, biomedicine, and energy storage, but with significant challenges in printability and particle dispersion to avoid agglomeration [31]. Zinc oxide nanoparticles, a well-known multifunctional material, has been used in VPP process to improve the printing resolution acting as substitutes of the photo-absorbing additives [32]. Results showed that ZnO nanoparticles have produced a more effective curing VPP process, which provides improved tensile strength, fracture strain, and Young’s modulus. Other researchers used silica micro silica spheres [33] to control the degree of curing in the VPP process, finding that the degree of curing increases with increasing filler fraction for a constant exposure dose of light, and that the geometric accuracy and surface roughness of printed samples decrease with a higher exposure dose and particle contents. The silica ceramic cores reinforced by mullite fibers, on the other hand, have been shown to improve strength and reduce porosity [34], while reducing shrinkage and increasing the creep resistance. Furthermore, silica coated with gold nanostructures have been developed for the formulation of more optically clear, printable, and photothermally responsive methacrylate resins for VPP process [35], reporting a recorded temperature enhancement of more than 80 °C per minute, for formulations containing 84 μg of gold by 1 g of resin using a 700 mW laser source.

The use of metal nanoparticles in the VPP process has been increasing in the last years [36], typically aiming for better mechanical properties, electrical conductivity, or thermal conductivity. Copper-reinforced nanocomposites have been used in applications that include microfluidics, biomedical devices, soft robotics, and drug delivery systems [37].

In biomedical applications, copper has been investigated for its antibacterial effects [36]. It has been found that nanocomposites inhibited cultures of bacteria such as the gram-positive *S. aureus*, but inconclusive positive results were obtained regarding the gram-negative *E. coli*.

Other research showed the use of copper–nickel nanowires using the VPP process, with positive results of these as reinforcements [38] with increases over 50% in hardness and storage modulus.

There is also research on the development of special resins able to generate in situ during the VPP process with silver nanoparticles [39], enabling not only a better reinforced nanocomposite but also a multifunctional material with potential applications in food packaging and biomedicine, as a consequence of the antimicrobial effects of silver nanoparticles. Other research has investigated the enhancement of VPP resins with silver nanoparticles [40], particularly higher thermal stability and improved heat transfer performance.

The following research investigated the tensile strength of VPP nanocomposites made with copper nanoparticles under different curing conditions, aiming to develop inexpensive technology to be used elsewhere. The main purpose of this material is to explore, with photopolymer resin and copper nanoparticles, inexpensive structural, biological, or electronic fabrication, particularly made by additive manufacturing, and targeting multifunctional applications. In particular, the study looked not only for mechanical properties, but also for their variability, which is important for the manufacturing of products at large-scale production.

## 2. Materials and Methods

The copper nanoparticles (Cu-NPs) were obtained from American Elements (Los Angeles, CA USA). The properties of the purchased Cu-NPs stated by the provider are described in Table 1; however, a morphological analysis of the purchased material was carried out to obtain the actual size of the used particles. The UV-photopolymer resin was purchased from Anycubic (from a local provider in Colombia). The resin used was a standard clear photosensitive resin with a curing UV wavelength of 365–405 nm and a density of 1.13 g/cm^3^.

**Figure 1:**
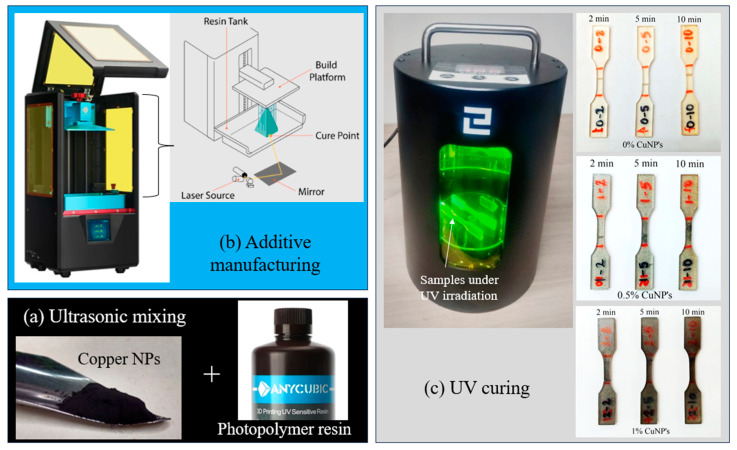
Overview of the nanocomposite fabrication method, (**a**) mixing processing, (**b**) additive manufacturing, and (**c**) UV curing post-processing.

The first step was to prepare two different samples of a Cu-NP–resin mixture containing 0.5% and 1% weight ratio of Cu-NPs. Then, the materials were mixed using a vortex mixer at 200 rpm for 2 h. A second mixing process was performed using an ultrasonic mixer for a total of 5 min. A sample with no Cu-NPs (0%) was also used for this experiment as a control sample. After completing the 2-stage mixing process, standard test specimens were printed using a 3D printer, a stereolithography (SLA)-type Anycubic photon mono. A graphical summary of the fabrication method can be seen in Figure 1, which shows Figure 1a mixing processing, Figure 1b additive manufacturing, and Figure 1c UV curing post-processing.

**Figure 2:**
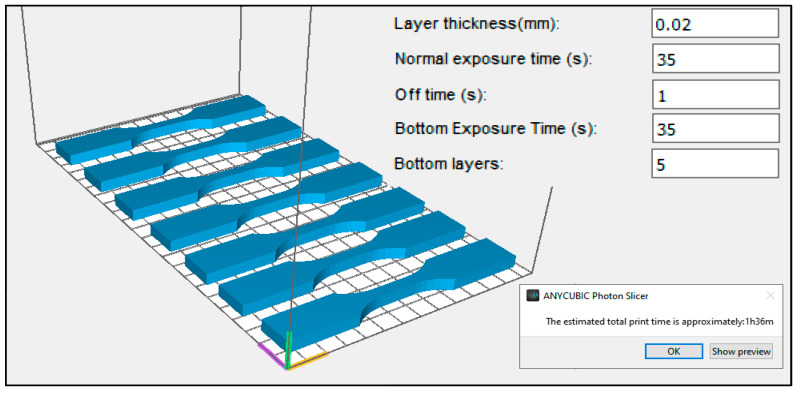
Printing parameters and preliminary view of test specimens.

Linear The printing parameters along with a preliminary view of the modeled specimens are shown in Figure 2.

A total of three specimens per sample (0%, 0.5%, and 1%) were printed, and a post-processing method was applied in the following way: each specimen was exposed to UV light in a chamber using three different post-curing times (2 min, 5 min, and 10 min). In this way, we obtained three different specimens for each sample with the intention to evaluate the influence of both Cu-NPs’ composition and post-curing times in the final properties of the obtained nanocomposite material. The irradiation exposure times were selected based on the following criteria: sustainable process (needs short processing times), sample deterioration (too much irradiation exposure increases the shrinkage of the sample and can turn the sample fragile), and able to produce visible changes (samples in fact changed color with the irradiation).

Shrinkage tests were performed on the samples, using a regular caliper, Mitutoyo-Japan, with a resolution of 0.01 mm. Three measurements were taken in the x, y, and z directions of the green bodies and then of the UV treated samples, for samples of 0.0% Cu-NPs and resin with 1% of copper nanoparticles (1.0% Cu-NPs) after 10 min UV curing.

**Figure 3:**
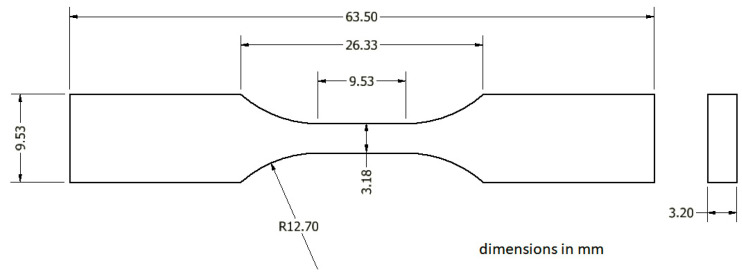
ASTM Type V test specimen dimensions.

The samples were tested under tensile tests in a Shimadzu Universal machine at room temperature. The dog-bone-shaped specimens were fabricated under the ASTM type V dimensions, and had a thickness of 3.20 mm, as shown in Figure 3.

The morphological analysis of the Cu-NPs was performed using scanning electron microscopy with energy dispersive X-ray spectroscopy (SEM-EDS) at the Universidad de Antioquia, Colombia. Samples were all gold sputtered before the observation. Several images (3) were analyzed to determine the particle distribution, which corresponds to the particle size of the Cu-NPs used.

In addition, a Weibull statistics analysis for the samples of 0.0% Cu-NPs and resin with 1% of copper nanoparticles (1.0% Cu-NPs) after 10 min UV curing was included to analyze the variability in the tensile strength property, as well as the values in these two compositions in a broader number of samples. Finally, a quick test of viscosity over the vat resin and the metal particles was performed at room temperature, with resin and particles premixed for 3 min. The tests were performed in a regular rotary viscometer.

## 3. Results and Discussion

**Figure 4:**
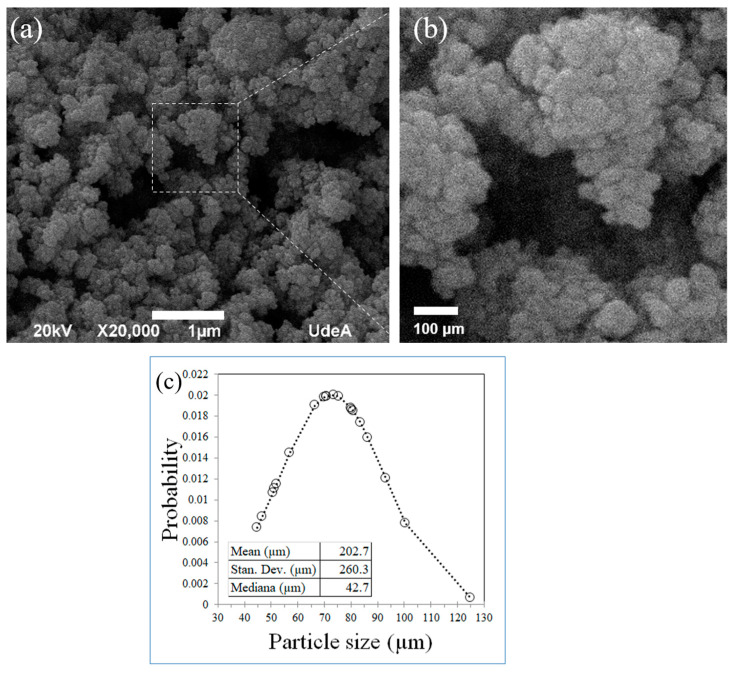
Morphological analysis of Cu-NPs: (**a**) SEM image at 20,000×, (**b**) detail at higher magnification, and (**c**) particle size distribution.

Figure 4 shows the scanning electron microscopy images for the Cu-NPs. Figure 4a was taken at 20,000×, and shows the NPs as provided by the seller, while Figure 4b is a detail of Figure 4a clearly showing that the particles were in the ranges of the nanoscale, all under 100 nm diameter. Figure 4c summarizes the particle size distribution obtained from the particle diameters measured in the SEM. The mean particle size was 72.9 nm. Very few particles over 100 nm were found in the images.

**Figure 5:**
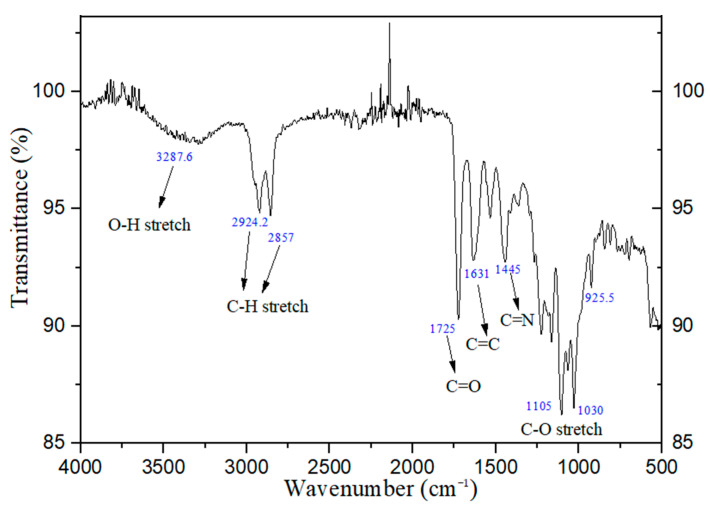
Fourier transform infrared spectroscopy (FTIR) for the UV-photopolymer resin used in this research.

Figure 5 shows Fourier transform infrared spectroscopy (FTIR) for the UV-photopolymer resin used in this research. The spectrum shows O-H and C-H single stretch types, and double bonds C=O, C=C, and C=N stretch types.

On the other hand, the viscosity of the formulation showed the following values: 0.4 ± 0.1 Pa.s for sample 0.0% Cu-NPs, 0.96 ± 0.12 Pa.s for sample 0.5% Cu-NPs, and 1.8 ± 0.15 Pa.s for sample 1.0% Cu-NPs. As noted, the viscosity values were increased significantly by the presence of the particles.

**Figure 6:**
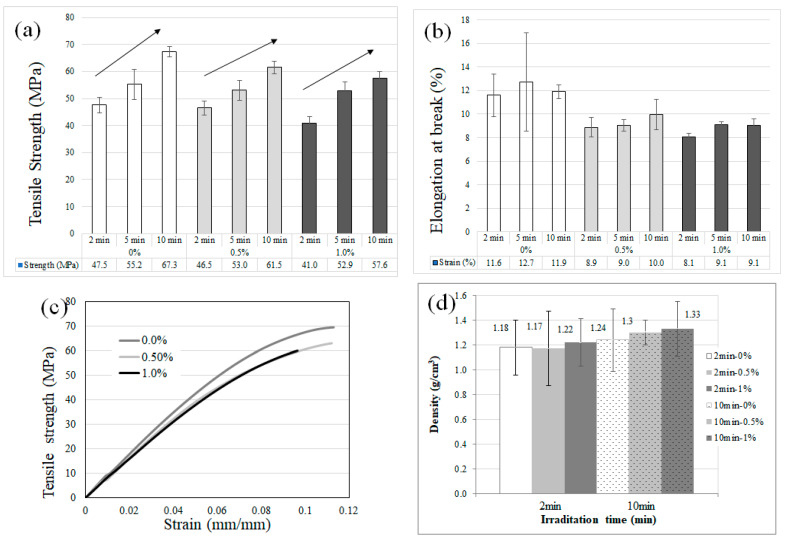
Tensile tests results for the polymer matrix nanocomposites made by additive manufacturing, (**a**) tensile strength, (**b**) elongation at break, (**c**) typical stress–strain curves; and (**d**) density results. The colors in figures (**a**,**b**) group the same particle loading in the sample.

Figure 6 summarizes all the mechanical characterization conducted in this research. Figure 6a shows the tensile strength for the different formulations after 3 different UV irradiation exposure times, 2, 5, and 10 min. A decrease in the mean values with the particle loading was observed, which is expected for this type of composite, since the particles can interrupt the stress continuity by stress concentration, and thus decrease the overall strength, particularly if there is some particle agglomeration and void derived in the process [41]. Additionally, there is a significant increase in the tensile strength with the increase in UV post-processing times, which is associated with an increase in the crosslinking process [42]. Figure 6b shows the elongation at break, which was reduced when particles were added, which, as explained before, is due to the particles generating load concentration points in the polymer matrix. It is also shown that elongation at break is improved with UV exposure in all cases. Figure 6c shows that the stress–strain curves have a typical behavior, with a fast failure once the main damages start, which can be further improved if fibers are used instead of particles, and by using dispersing chemicals that decrease particle precipitation/agglomeration. The elastic modulus was estimated from these data, giving 1.0 ± 0.2 GPa for sample 0% Cu-NPs, 0.67 ± 0.3 GPa for sample 0.5% Cu-NPs, and 0.55 ± 0.1 GPa for sample 1.0% Cu-NPs.

Figure 6d shows density tests for the samples neat resin (0.0% Cu-NPs) and resin with 1% of copper nanoparticles (1.0% Cu-NPs) after 2 and 10 min UV curing. Results showed that the density is basically the same, but in general it is a bit higher for samples with the nanoparticles, and slightly increases with the UV curing, which is related to compounds’ release and shrinkage.

**Figure 7:**
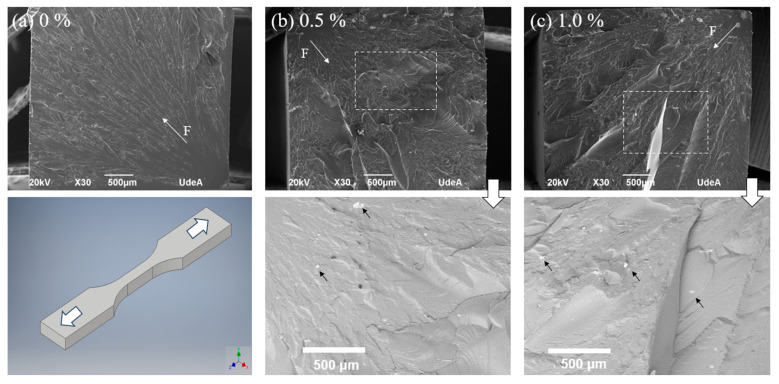
SEM (secondary electrons) images of the fractures from tensile tests for the polymer nanocomposites with different copper nanoparticle loading: (**a**) 0% Cu-NPs, (**b**) 0.5% Cu-NPs, and (**c**) 1.0% Cu-NPs. The arrows are magnifications of the corresponding images taken with backscattered electrons.

Figure 7 shows SEM images for typical fractured surfaces of the samples. In all cases, the fracture direction and the damage initiation point are easily identified (white arrows). Figure 7a in general shows a smoother fracture for the neat resin sample; this is 0.0 wt % Cu-NPs, when compared to other samples. The particle additions in Figure 7b,c produce a more irregular fracture surface, which can be associated with crack growing directed by the particles, which of course is not necessarily in the same plane, therefore producing more irregularities. The details for Figure 7b,c in the bottom of the image show the areas where particles were conglomerated (black arrows). These images were taken with the backscattered electrons detector. As has been reported before, particle additions in tensile conditions can be detrimental to strength and ductility [43], which is supported by these results, where the failure is not only catastrophic (see Figure 7c), but also the fracture surfaces are relatively flat. However, in many cases, the adhesion between the particles and the resin can be enhanced, and therefore the strength of the composite- and strain-derived properties can also be increased [44] up to an optimal value.

**Figure 8:**
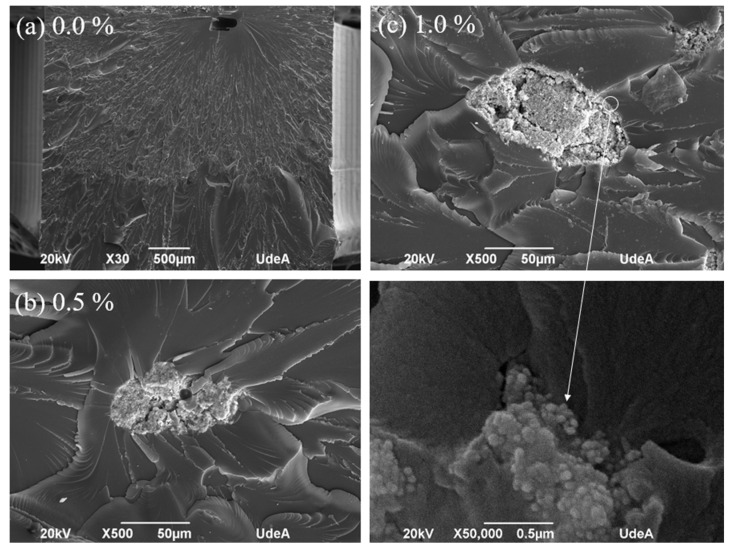
Details obtained by SEM from some of the issues found in some of the 3D printed samples: (**a**) 0.0% Cu-NPs, (**b**) 0.5% Cu-NPs, and (**c**) 1.0% Cu-NPs.

Figure 8 shows details of some of the microstructural defects found in the samples, revealed by SEM. To clarify, these defects are not the general case, as most samples did not show them, but they are important for analysis and further improvement of the manufactured parts. In a few samples some voids appeared; see Figure 8a for a sample with 0.0 wt % Cu-NPs, which might be investigated and optimized via 3D printing process parameters, as these voids cause a significant decrease in mechanical strength. Figure 8b shows particle agglomeration for a sample with 0.5 wt % Cu-NPs. In some cases, particles were agglomerated, and this defect also significantly decreased the bulk properties of the nanocomposite. Figure 8c shows a sample with 1.0 wt % Cu-NPs with particle agglomeration with the same issues for strength. However, the details show a very good resin impregnation, and confirm the nanoparticle size reinforcements added. These issues can also be improved by optimizing the mixing stage and improving the manufacturing process in general.

**Figure 9:**
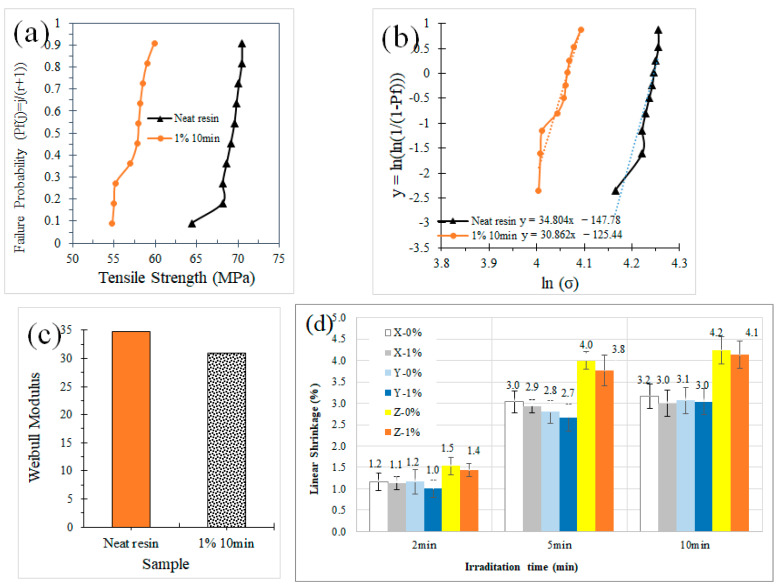
Weibull statistics comparing tensile strength for the neat resin and sample with 1% of copper NPs: (**a**) failure probability, (**b**) data fit, (**c**) Weibull modulus, and (**d**) linear shrinkage tests.

Finally, Figure 9 summarizes the variability of samples in different tests. First, Weibull statistics are given for the samples neat resin (0.0% Cu-NPs) and resin with 1% of copper nanoparticles (1.0% Cu-NPs) after 10 min UV curing. The curve was built with 10 tensile tests over 10 samples per material formulation. Figure 9a shows the tensile strength with the failure probability, confirming that particles decrease tensile strength, because they are acting as stress concentrators. Figure 9b shows the linear fit to obtain the Weibull modulus from Figure 9c, in which the values are 34.8 and 30.9 for neat resin and 1.0% Cu-NPs, respectively, which are very high considering they are 3D printing samples, and that this number accounts for a very low variability, since as the modulus increases the variability in the tensile strength property decreases, meaning that the used materials are repetitive and reliable in the results. Additionally, variability was accounted for via shrinkage tests for samples after 2, 5, and 10 min of UV curing. Results showed a low shrinkage as expected for this technology, all below 5%. Samples with the particles showed lower shrinkage, which is related to the filler itself being more resistant to the temperature process, as copper does not lose weight during the irradiation process. Samples irradiated for 5 and 10 min basically showed the same values for the same coordinate, which means after 5 min not many changes were produced in the dimensions. These results were not very different to those obtained in the tensile tests. Additionally, X and Y directions were similar in values, while Z showed much larger values, which was expected as the layers’ interface is in this coordinate, which introduces more defects and voids in Z.

## 4. Discussion

This investigation has shown the effective fabrication of polymer matrix nanocomposites made via vat photopolymerization technology (VPP), composed of a UV photopolymerization resin and up to 1.0 wt % of copper nanoparticles (Cu-NPs). The process involved UV post-processing up to 10 min, which produced improved tensile strength. This method itself was performed as described above because it involved a very inexpensive machine, a stereolithography (SLA)-type Anycubic photon mono, which currently can be as inexpensive as 280 USD, while the copper nanoparticles used were not further chemically treated, and the post-curing chamber was less than 100 USD. This is an important condition of the process to guarantee the solution can be scalable and successfully implemented in many countries or for small scale, such as for individuals or small business enterprises. Since manufacturing is known to be very fast and with low waste of parts and defects, the process can be further implemented for industry. The developed material showed a consistently very high Weibull modulus, corresponding to low variability, and far beyond the variable construction materials, with very low modulus and thus highly variable properties. Additionally, the tensile values, besides the particles, were very competitive, all over 40 MPa, and responded well to the irradiation treatment by improving strength and maintaining consistent density values. This validates the inexpensive process as a potential approach for electronics, structural applications, or biomedicine with additive manufacturing.

In general, it has been found that adding these nanoparticles improves tensile strength, and elongation at the break slightly decreases with the particle contents, as expected for this type of reinforcement [45,46,47] (in which, under many conditions, the particles can act as stress concentrators and diminish the tensile strength), but also that, with the curing time, these properties can be further improved. However, it is also expected that these particles are, as shown in many studies, much better under compression loadings [48,49], as the crack growth is not favored as in tensile conditions. The tensile strength could also be improved by combining copper nanoparticles and fibers, such as ceramic fibers (carbon and glass), or even their metallic counterparts, which can produce the complexity of worsening properties for 3D printing such viscosity [50] and optical parameters needed for the resin during the manufacturing, particularly the curing process [51] during the VPP process by the UV light.

As mentioned above, the application of VPP processes of nanocomposites with metal nanoparticles is an intense field of research, particularly because the particles not only can improve mechanical properties, thermal stability, and antimicrobial effects [36,38,39,40], but can also be used conductive polymers with high particle loading, and perhaps microfibers that facilitate percolation for better electrical conductivity [52]. In particular, it was shown that this polymer–silver nanocomposite is significantly affected by agglomeration: slightly agglomerated silver particles produced a resistivity of 2.0 × 10^−1^ Ω cm, while for highly agglomerated composite the value was 2.9 × 10^3^ Ω cm (silver content of 20 vol.%.) Low contents of silver particles produced a resistivity of 5.6 × 10^5^ Ω cm. On the contrary, hybrid additive manufacturing mixing vat photopolymerization and laser-activated selective metallization for three-dimensional conformal electronics has produced highly conductive nanocomposites [53]: with a thickness of plated copper layer of 11 µm, the electrical conductivity was 5.2 × 10^7^ S/m, very close to pure copper (5.8 × 10^7^ S/m). Thus, hybrid additive manufacturing for electronics, including particles, is a promising area for several reasons that include feasible manufacturing and materials and improved properties. These are areas to explore, for instance, in countries where the electronics industry is starting, which can give an advantage over complex and expensive solutions, as is the case in Latin America.

Finally, it is important to note that the particles increased significatively the viscosity, which can limit the fabrication; however, the obtained values were of the same order of magnitude as reported for a similar resin but with Ag particles [54]. During the manufacturing, precipitation of the Cu-NPs to the bottom of the tank of the printer was observed, which promotes the agglomeration of the particles. These agglomerated spots become caught in the bottom layers of the printed part, thus generating starting points for early fractures, and finally reducing the mechanical properties of the final material. However, a series of improvements in the manufacturing process can be made to achieve much better results and obtain a nanocomposite material with higher mechanical properties than the matrix itself. For instance, to achieve a better degree of resin impregnation on the Cu-NPs, pre-processing of the particles must take place. Such processes can include chemical processing or heating methods to improve the adherence of the Cu-NPs to the resin. To avoid precipitation, and hence agglomeration of the particles at the bottom layers of the printed part, it is recommended to use a dispersion emulsifier on the resin that allows the nanoparticles to stay suspended in the mixture.

## 5. Conclusions

This research showed photo resin nanocomposites made by UV-photopolymerization additive manufacturing with copper nanoparticles. In tensile tests, the particle loading decreased the strength, while the UV irradiation exposure increased the strength values. The Weibull statistics revealed a very high modulus, which is directly associated with a low property (tensile strength) variability, which is very useful in manufacturing and product qualification.

As expected with nanomaterials, the inclusion of nano-sized particles in a photo resin generated very significant effects, which were very clear in the changes of the mechanical properties, even at the low particle loading used in this research.

Despite obtaining a reduction in the mechanical properties of the material with the increase of Cu-NPs, such precipitation and particle agglomeration in specific layers of the final material enable the material to be a candidate for improving the electrical conductivity at specific layers of a nanomaterial, and open the door for future research regarding electrical conductivity in several layers of nanocomposite materials obtained with vat photopolymerization methods.

## Figures and Tables

**Table 1:** Properties of Cu-NPs provided by American Elements.

Property	Reported
Molecular weight	63.55
Appearance	Black-Brown
Size range	2–90 nm
Particle size	25 nm
Specific surface area	30–50 m^2^/g
Morphology	Spherical
Poisson’s Ratio	0.34
Vickers Hardness	369 MPa

## Data Availability

Data will be available upon request to the contact author. The data are not publicity available due to privacy.
